# Succession Influences Wild Bees in a Temperate Forest Landscape: The Value of Early Successional Stages in Naturally Regenerated and Planted Forests

**DOI:** 10.1371/journal.pone.0056678

**Published:** 2013-02-15

**Authors:** Hisatomo Taki, Isamu Okochi, Kimiko Okabe, Takenari Inoue, Hideaki Goto, Takeshi Matsumura, Shun'ichi Makino

**Affiliations:** 1 Department of Forest Entomology, Forestry and Forest Products Research Institute, Tsukuba, Ibaraki, Japan; 2 Forestry and Forest Products Research Institute, Tsukuba, Ibaraki, Japan; 3 Tama Forest Science Garden, Forestry and Forest Products Research Institute, Hachioji, Tokyo, Japan; 4 Kyushu Research Centre, Forestry and Forest Products Research Institute, Kumamoto, Kumamoto, Japan; 5 Nasushiobara, Tochigi, Japan; University of Northampton, United Kingdom

## Abstract

In many temperate terrestrial forest ecosystems, both natural human disturbances drive the reestablishment of forests. Succession in plant communities, in addition to reforestation following the creation of open sites through harvesting or natural disturbances, can affect forest faunal assemblages. Wild bees perform an important ecosystem function in human-altered and natural or seminatural ecosystems, as they are essential pollinators for both crops and wild flowering plants. To maintain high abundance and species richness for pollination services, it is important to conserve and create seminatural and natural land cover with optimal successional stages for wild bees. We examined the effects of forest succession on wild bees. In particular, we evaluated the importance of early successional stages for bees, which has been suspected but not previously demonstrated. A range of successional stages, between 1 and 178 years old, were examined in naturally regenerated and planted forests. In total 4465 wild bee individuals, representing 113 species, were captured. Results for total bees, solitary bees, and cleptoparasitic bees in both naturally regenerated and planted conifer forests indicated a higher abundance and species richness in the early successional stages. However, higher abundance and species richness of social bees in naturally regenerated forest were observed as the successional stages progressed, whereas the abundance of social bees in conifer planted forest showed a concave-shaped relationship when plotted. The results suggest that early successional stages of both naturally regenerated and conifer planted forest maintain a high abundance and species richness of solitary bees and their cleptoparasitic bees, although social bees respond differently in the early successional stages. This may imply that, in some cases, active forest stand management policies, such as the clear-cutting of planted forests for timber production, would create early successional habitats, leading to significant positive effects for bees in general.

## Introduction

In many temperate terrestrial forest ecosystems, natural disturbances such as avalanches, windstorms, fires, and floods, and human disturbance including tree harvesting, drive the reestablishment of forests [1]. Succession in plant communities, in addition to reforestation following the creation of open sites through harvesting or natural disturbances, can affect forest faunal assemblages. For example, the intermediate disturbance hypothesis suggests that shifts in species richness are likely and the midway stage of succession would contain the highest species richness [2], [3]. Previous studies have consistently demonstrated that the species richness of various taxa peak at different times as succession proceeds, indicating that the diverse taxa within a community include species that prefer specific successional stages [4], [5]. Among the wide range of forest successional stages, the conservational and ecological importance and the value of the early stages of succession may require more attention [6]. The early successional stage can be characterized by high productivity of plants, compound floral and faunal food webs, and large nutrient changes [6]. Previous studies have shown that this stage in a temperate forest region can have a great diversity in abundance and species richness of flora and fauna [7], [8].

Wild bees perform an important ecosystem function in human-altered and natural or seminatural ecosystems as bee species are important pollinators for both crops and wild flowering plants [9]. Pollinators including wild bees can increase the production of approximately 75% of the 115 most essential crops worldwide [10], and the proportion of flowering plants pollinated by animals is estimated to be 87.5% of all flowering plants [11]. The conservation and creation of seminatural and natural land cover at optimal successional stages for wild bees are important to maintain a high abundance and species richness for pollination services [12], [13]. Examples of this include human-dominated landscapes with a long history of human land use, among which grasslands are a notable seminatural habitat. One study on wild bees, covering a period of 1 to 5 years of secondary succession of European fallow land, found the highest species richness after 2 years [14]. However, another study of wild bees in European limestone quarries found little difference in abundance and species richness among habitats ranging from 1 to 121 years old [15]. This indicates that even newly created and permanent grassland-like habitats provide important habitats for wild bees

In regions dominated by temperate forests, bee abundance and species richness within the forest area are known to increase with decreasing forest cover in the surrounding landscape [16]. This finding suggests that even if an optimal habitat for bees were created, a rapid decrease in abundance and species richness would occur with habitat succession in regions where forest regeneration to canopy closure occurs rapidly from grassland-like habitats. Rapid forest regeneration to canopy closure of matured and old-growth forest provides a habitat for closed-forest wildlife but presents a trade-off with early successional species [6], [8]. Therefore, optimal management of the successional stages of seminatural habitat would help to maintain high abundance and species richness of bees at regional and landscape scales, providing effective pollination services.

We aimed to determine the optimal successional stages, and to propose better management practices of seminatural habitat, for wild bees in a temperate forest region, in order to ensure pollination services in the region. We examined the effects of reforestation stages, from recently harvested to old growth forests between 1 and 178 years old. Bee assemblages were examined for the two different reforestation types of planted conifer and naturally regenerated forests. Plant species composition regularly varies between different types and ages of reforestation [17]. Here, we tested the hypothesis that the different successional stages of seminatural habitats would affect the abundance and species richness of wild bees, but the responses of wild bees might differ depending on the characteristics of the bees (i.e., social or parasitic status). We particularly assessed the importance of the early successional stages for bees, which has been suspected but not previously proven [18]. We conducted this study in Japan, where the most intensive anthropogenic reforestation efforts involve the conversion of natural or seminatural broad-leaved forests to monocultures of conifer trees. To accommodate the high demand for timber after the Second World War, broad-leaved trees were harvested and coniferous species, primarily *Cryptomeria japonica*, *Chamaecyparis obtusa*, and *Larix leptolepis*, were planted until the 1970s. Such conifer plantations now account for approximately 40% of the total forest cover in Japan [19].

## Materials and Methods

### Study Region and Sites

**Figure 1:**
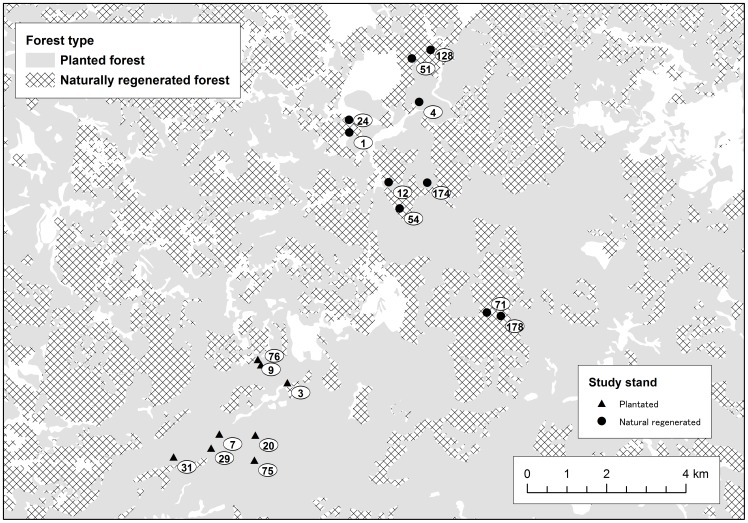
The study region in the northern part of Ibaraki Prefecture in central Japan (approximately 36°50′–7′N, 140°32′–8′E; 500–50 m above sea level) and locations of the study stands within areas of planted conifers and naturally regenerated forests. Numbers indicate reforestation age.

The study region was the northern part of Ibaraki Prefecture in central Japan (approximately 36°50′–7′N, 140°32′–8′E; 500–50 m above sea level). The annual mean temperature of the study region is 10.7°C, and the mean annual precipitation is 1910 mm [20]. The landscapes have undergone dramatic anthropogenic changes since the Second World War. Intensive planted reforestation conducted in this area has converted the landscape from natural or seminatural forests of broad-leaved trees, dominated by *Quercus serrata*, *Quercus mongolica*, and *Fagus crenata* to monoculture conifer plantations of *Cryptomeria japonica* and *Chamaecyparis obtusa* [21]. For this study, 10 naturally regenerated deciduous forest stands (2.53–32.49 ha) and eight planted conifer (*C. japonica*) forest stands (2.55–14.29 ha) were selected (Table 1; Figure 1). The ages of the naturally regenerated forest stands varied between 1 and 178 (1, 4, 12, 24, 51, 54, 71, 128, 174, and 178) years old after harvest. The ages of planted forest stands varied between 3 and 76 (3, 7, 9, 20, 29, 31, 75, and 76) years old after harvest. The conifer plantation trees in the study region are usually harvested at around 80 to 90 years of age. The ages were based on a tentative estimation of the National Forest of Japan.

**Table 1:** Study sites, 10 naturally regenerated and 8 conifer (*Cryptomeria japonica*) planted forest stands, in the northern part of Ibaraki Prefecture in central Japan (approximately 36°50′–57′N, 140°32′–38′E; 500–850 m above sea level).

ID	Reforestation type	Age	Area (ha)
N1	Natural	1	2.53
N4	Natural	4	4.91
N12	Natural	12	4.13
N24	Natural	24	23.56
N51	Natural	51	10.37
N54	Natural	54	14.72
N71	Natural	71	19.11
N128	Natural	128	32.49
N174	Natural	174	11.75
N178	Natural	178	9.29
C3	Conifer planted	3	4.28
C7	Conifer planted	7	6.04
C9	Conifer planted	9	4.9
C20	Conifer planted	20	4.88
C29	Conifer planted	29	14.29
C31	Conifer planted	31	12.07
C75	Conifer planted	75	3.13
C76	Conifer planted	76	2.55
	Mean	52.61	10.28

Stand ages were based on tentative estimations recorded in the National Forest Inventory of Japan.

### Bee Collection

We collected wild bees using standard Townes-type Malaise traps (Golden Owl Publishers; 180 cm long, 120 cm wide, and 200 cm high) [22] in 2002 for naturally regenerated forests and 2003 for planted conifer forests. In each of the selected forest stands, two traps were placed approximately in the center of the forest stand to avoid forest edge effects. A mixture of ethanol and propylene glycol was used to preserve bees captured in the Malaise traps. Trapped wild bees were collected every 2 weeks from late April to early November in both forest types. We brought all collected bees into the laboratory at the Forestry and Forest Products Research Institute, Tsukuba, where voucher specimens were kept, and identified at the species level. We then classified cleptoparasitic *Coelioxys*, *Epeolus*, *Nomada*, and *Sphecodes* species as “cleptoparasitic,” social *Apis* and *Bombus* species as “social,” and the remaining species as “solitary.” Although there may have been some species of primitively eusocial bees in the “solitary” group, such as species in the family Halictidae [23], we tentatively classified them as solitary because of the limited available information on their biology.

### Data Analysis

The effects of the successional stage of reforestation for both forest types on the abundance and species richness of bee groups (all, social, solitary, and cleptoparasitic bee assemblages) were determined using ordinary least-squares regression models. We conducted simple and quadratic functions because scatterplots used previously indicated that some results might have nonlinear effects from the successional stages. We disqualified results from the quadratic functions when the vertexes placed within the forest ages (between 3 and 76 years old for planted forest and between 1 and 178 years old for naturally regenerated forest) indicated values below 0 on the y axis, which were biologically illogical situations because bee abundance and species richness became minus values. As well as the simple linear and quadratic regression models, we examined models in which the successional stages of reforestation were square-root and log transformed. We then compared the models using Akaike's information criterion (AIC), the ΔAIC values obtained by the difference from AIC values of the null models. All of the analyses were performed using the R software (version 2.15.0) [24]. Prior to these regression analyses, we used Mantel tests to check for significant spatial autocorrelation among the study sites for the abundance and species richness of bees in naturally regenerated and planted conifer forest stands; no autocorrelation was found (all *p*>0.400). The ade4 package of R, with the number of permutations set to 1000, was used for the Mantel tests [25].

## Results

**Figure 2:**
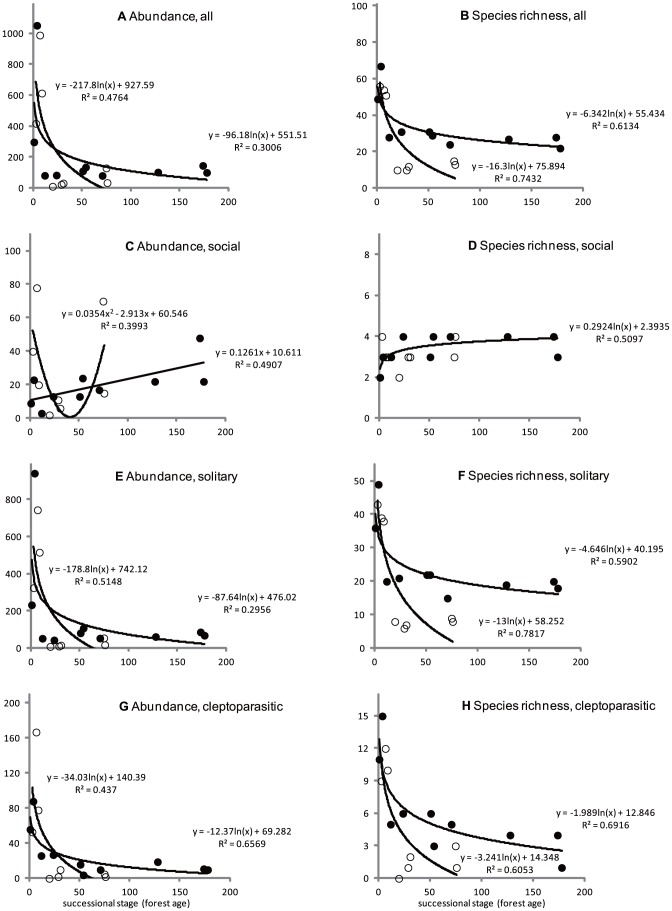
Effects of different successional stages on the abundance and species richness of bees, showing total (A and B), social (C and D), solitary (E and E), and cleptoparasitic (G and H) assemblages, in naturally regenerated and conifer (*Cryptomeria japonica*) planted forests. Y axes indicate the numbers of collected individuals for abundance and the number of collected species for species richness, respectively. Solid circles represent naturally regenerated forest, and open circles represent planted conifer forest.

We captured and identified a total of 4465 wild bee individuals from 113 species, representing 4 social (*Apis cerana*, *Bombus ardens*, *Bombus diversus*, and *Bombus hypocrita*), 88 solitary (32 *Andrena*, 6 *Ceratina*, 4 *Colletes*, 1 *Halictus*, 8 *Hylaeus*, 28 *Lasioglossum*, 4 *Megachile*, 1 *Macropis*, 2 *Osmia*, 1 *Tetralonia*, and 1 *Xylocopa*), and 21 cleptoparasitic (2 *Coelioxys*, 1 *Epeolus*, 13 *Nomada*, and 5 *Sphecodes*) species. Model comparisons for the naturally regenerated forest indicated that the simple linear regression model be selected for the abundance of social bees with the log-transformed regression models selected for the other categories (Table 2). Model comparisons for the planted conifer forest indicated that the lowest AIC value was obtained from the null model of species richness of social bees, and the quadratic regression model was selected for the abundance of social bees, with the log-transformed regression models selected for the other categories (Table 3). The effects of succession on all bees, solitary bees, and cleptoparasitic bees for both forest types exhibited similar trends. Higher abundances and species richness were observed in the early successional stages than in mature successional stages, and higher species richness was observed in naturally regenerated forest than in planted conifer forest as the successional stages progressed (Figure 2). However, the trends of successional effects on social bees were different. Higher abundance and species richness of social bees in naturally regenerated forest were observed as the successional stages progressed, whereas the abundance of social bees in planted conifer forest produced a concave-shaped relationship when plotted (Figure 1).

**Table 2:** Model comparisons, for the abundance and species richness of bee groups (all, social bees, solitary bees, and cleptoparasitic assemblages) in natural regenerated forest.

	All bees				Social				Solitary				Cleptoparasitic			
	abundance		species richness		abundance		species richness		abundance		species richness		abundance		species richness	
	AIC	ΔAIC	AIC	ΔAIC	AIC	ΔAIC	AIC	ΔAIC	AIC	ΔAIC	AIC	ΔAIC	AIC	ΔAIC	AIC	ΔAIC
null	145.369		83.856		81.285		24.169		143.676		78.018		96.530		59.459	
y = x	145.765	0.396	81.645	−2.211	76.538	−4.747	24.399	0.230	143.990	0.314	76.544	−1.474	94.218	−2.312	55.145	−4.314
y = x+x^2^	145.451	0.082	79.816	−4.040	78.338	−2.947	20.803	−3.366	143.874	0.198	74.005	−4.013	90.397	−6.133	54.247	−5.212
y = log(x)	143.794	−1.575	76.352	−7.504	79.870	−1.415	19.041	−5.128	142.171	−1.504	71.097	−6.921	87.832	−8.698	49.696	−9.763
y = sqrt(x)	144.667	−0.702	79.100	−4.756	77.671	−3.614	22.573	−1.596	142.928	−0.748	74.106	−3.912	91.175	−5.355	52.173	−7.286

The table shows values of AIC and of ΔAIC calculated as the difference from AIC values of the null models.

**Table 3:** Model comparisons for the abundance and species richness of bee groups (all, social bees, solitary bees, and cleptoparasitic assemblages) in conifer (*Cryptomeria japonica*) planted forest.

	All bees				Social				Solitary				Cleptoparasitic			
	abundance		species richness		abundance		species richness		abundance		species richness		abundance		species richness	
	AIC	ΔAIC	AIC	ΔAIC	AIC	ΔAIC	AIC	ΔAIC	AIC	ΔAIC	AIC	ΔAIC	AIC	ΔAIC	AIC	ΔAIC
null	119.883		74.847		79.729		18.516		116.102		70.825		90.871		50.644	
y = x	118.666	−1.216	72.215	−2.631	81.719	1.989	20.146	1.630	114.341	−1.761	67.709	−3.115	89.577	−1.294	48.900	−1.743
y = x+x^2^	115.345*	−4.538	58.538*	−16.309	79.653	−0.077	18.751	0.235	110.824*	−5.278	51.292*	−19.533	88.165*	−2.706	42.407*	−8.236
y = log(x)	116.706	−3.176	65.972	−8.875	81.418	1.689	20.431	1.915	112.316	−3.786	60.649	−10.176	88.276	−2.595	45.207	−5.437
y = sqrt(x)	117.410	−2.472	69.494	−5.353	81.683	1.953	20.463	1.947	112.973	−3.129	64.698	−6.126	88.590	−2.281	47.018	−3.626

The table shows values of AIC and of ΔAIC calculated as the difference from AIC values of the null models. Asterisks (*) indicate that the vertexes placed within the forest ages (between 3 and 76 years old for planted conifer forest) were below 0 on the y axis for the best models (quadratic functions), and thus the second-best models were selected.

## Discussion

The response of all bees to the different successional stages of naturally regenerated and planted conifer forests indicated that the early successional stages provide important habitats for bees. The majority of collected bees were solitary species. Cleptoparasitic species do not collect pollen and nectar for their offspring and were parasites of the solitary bees collected in the study. Therefore, the trends for all bees may be based on the trends observed for the solitary and cleptoparasitic bees. Although there are several bee species that might depend on habitats provided during the late successional stages of temperate forests [26], it is suspected that most bees prefer open and disturbed habitats to late successional stages [18], [23]. Solitary bees have specific habitat requirements and are considered to be more restricted to grassland-like habitats than social bees [27], [28]. Forestry practices such as clear-cutting and thinning offer a wide variety of habitat transitions but frequently create more open and early successional habitats, which generate more floral and habitat resources for bees [8]. This implies that many bee species would respond positively to forest management practices in a region where forest regeneration to canopy closure occurs quickly, such as our selected study region [22]. Our results also demonstrated that abundance and species richness were as high in the early successional stage of planted conifer forest as in naturally regenerated forest. If such young planted forests can provide suitable habitats for bees, even in a forest managed for timber production, active clear-cutting of planted forests that generates early succession will have substantial positive effects on early successional species [8].

For example, in Japan, the area of grassland and young forests has declined rapidly [29] to the extent that 68% of the national land area is covered by forest, of which 42% is plantation [30]. Forestry activities in Japan declined after the 1960s and 1970s with the increase in imported timber following international free trade agreements and the introduction of a floating exchange rate system [31]. Many of the trees in planted forests are now reaching harvest age, although the wood supply from the forests has slightly increased [29]. As the area of grassland and early successional forests in Japan has declined, a national decline in bird and butterflies species, which are dependent on early successional stages, is also thought to have occurred [32], [33]. Trade-offs must be carefully considered when planning the extent and location of forest cutting. These trade-offs can be between bee diversity and other taxonomic diversity, or between pollination and other ecosystem services [34], such as carbon storage, flood control, water provision, and timber production, which are provided by mature naturally regenerated and planted forests. Some active clear-cutting of planted forests, leading to early succession, might become an acceptable policy in Japan, although conservation of primary growth forests and careful rotation planning of where to cut, at both a landscape and national scale, are required.

Wild bees pollinate various crops [35]–[40]. The area surrounding bee-pollinated crop fields would ideally be a grassland-like habitat, and hence the management and quality maintenance of seminatural and natural ecosystems around agricultural fields are important [41], [42]. For the example of almond production in California, it was found that practices within farms, such as organic farming alone, would not sustain pollination services by wild bees, and the presence of high-quality habitats along the edges of the orchards are important [43]. In many temperate terrestrial forest ecosystems, forest regeneration to canopy closure occurs quickly; hence the creation and maintenance of early successional stages of both naturally regenerated and planted conifer forests, surrounding bee-pollinated cropland could help to enhance pollination services. For example, near the present study region, in the northern part of Ibaraki Prefecture in central Japan, common buckwheat, which is a heterostylous crop that depends highly on insect pollination, is a distinctive crop grown by local landholders in mountainous regions [39], [44]. Such a crop might benefit from the habitats created for bees.

Although the species richness of the social bees collected was relatively low (four species), our results indicated that the responses of social bee species to the different forest successional stages were different from those of solitary and cleptoparasitic bees. As the successional stages progressed, positive relationships were observed for the abundance and species richness of social bees in naturally regenerated forest. However, as successional stages progressed, the convex relationship for abundance and no relationship for the species richness of social bees were observed in planted conifer forest. In naturally regenerated forest, nesting resources might be more widely available in mature successional stages than in early successional stages. This could include cavities in large trees for *Apis cerana* [45], [46] and underground cavities for the *Bombus* species [47]. In planted conifer forests, the early succession stages may provide floral resources for the *Apis* and *Bombus* bees; on the other hand, late successional stages (e.g., the 75- and 76-year-old stands) may have lower floral resources but more underground cavities for nesting sites for *Bombus* species. Planted forests are usually disturbed by active human management, such as pre-commercial tree thinning and understory weed clearing [48]; thus the 20- and 31-year-old forest stands might have contained fewer nesting sites for the *Bombus* species as well as poorer floral resources. It is important to interpret our results considering the effects of wider spatial scales on *Apis* and *Bombus* species [49]. The foraging ranges of these social bees are likely wider than those of the majority of the solitary bees [50]–[52]. Previous studies have indicated that *Apis cerana* forages over 1 km [53] and the spatial ranges of landscape effects, which influenced its foraging, were from 1 to 3 km [39], [41]. Although the foraging ranges of our captured *Bombus* species were unknown, other *Bombus* species have been known to forage for at least 250 m to 1.5 km [54]–[57]. Therefore, influences at the landscape level rather than at the forest stand level might have influenced the responses of these social bees.

Wild bees were sampled during a single year in each reforestation type using Malaise traps in the present study. There was a risk that this particular sampling method may be biased for or against particular groups of bees, and may not represent sampling completeness of the bee fauna in the study region. However, we sampled 4465 wild bee individuals from 113 species using the Malaise traps. Based on the sampled bees, we can conclude that the early successional stages of both naturally regenerated and planted conifer forests maintain high abundances and species richness of solitary bees and their cleptoparasitic bees, although social bees respond differently in early successional stages. These results may have implications for active forest stand management practices, such as clear-cutting of planted forests for timber production, which would create early successional habitats, leading to significant positive effects on bees in general. However, in addition to the conservation of primary, old growth natural forests, trade-offs between bee diversity and other taxonomic diversity in relation to pollination and other ecosystem services should be considered.

## Supporting Information

Table S1 **List of bee species and the number of individuals sampled in each forest stand.**
(DOC)
