# Supplementary material for: Succession Influences Wild Bees in a Temperate Forest Landscape: The Value of Early Successional Stages in Naturally Regenerated and Planted Forests
Source: PLoS One. 2013 Feb 15;8(2):e56678. doi: 10.1371/journal.pone.0056678 (PMC3574003; doi:10.1371/journal.pone.0056678)
Supplement: Table S1 — List of bee species and the number of individuals sampled in each forest stand. (DOC) [file pone.0056678.s001.doc]

Table S1. List of bee species and the number of individuals sampled in each forest stand.

| **Species** | **Class** | **N1** | **N4** | **N12** | **N24** | **N51** | **N54** | **N71** | **N128** | **N174** | **N178** | **C3** | **C7** | **C9** | **C20** | **C29** | **C31** | **C75** | **C76** |
| --- | --- | --- | --- | --- | --- | --- | --- | --- | --- | --- | --- | --- | --- | --- | --- | --- | --- | --- | --- |
| *Apis cerana* | social | 7 | 0 | 1 | 2 | 3 | 5 | 1 | 1 | 1 | 2 | 10 | 0 | 1 | 0 | 0 | 0 | 0 | 1 |
| *Bombus ardens* | social | 0 | 11 | 0 | 2 | 8 | 2 | 9 | 10 | 19 | 18 | 1 | 21 | 3 | 1 | 5 | 1 | 20 | 1 |
| *Bombus diversus* | social | 0 | 11 | 1 | 7 | 0 | 9 | 4 | 7 | 12 | 2 | 27 | 48 | 16 | 1 | 5 | 3 | 37 | 6 |
| *Bombus hypocrita* | social | 2 | 1 | 1 | 2 | 2 | 8 | 3 | 4 | 16 | 0 | 2 | 9 | 0 | 0 | 1 | 2 | 13 | 7 |
| *Andrena akitsushimae* | solitary | 1 | 0 | 1 | 2 | 0 | 2 | 9 | 3 | 8 | 1 | 16 | 35 | 11 | 1 | 5 | 0 | 8 | 3 |
| *Andrena benefica* | solitary | 0 | 3 | 0 | 0 | 0 | 1 | 0 | 2 | 2 | 1 | 0 | 2 | 1 | 0 | 0 | 0 | 0 | 0 |
| *Andrena brevihirtiscopa* | solitary | 0 | 0 | 0 | 0 | 0 | 1 | 0 | 0 | 0 | 0 | 0 | 0 | 0 | 0 | 0 | 0 | 0 | 0 |
| *Andrena dentata* | solitary | 0 | 1 | 0 | 0 | 0 | 0 | 0 | 0 | 0 | 0 | 0 | 0 | 1 | 0 | 0 | 0 | 0 | 0 |
| *Andrena hebes* | solitary | 1 | 14 | 1 | 1 | 4 | 15 | 3 | 3 | 3 | 0 | 4 | 51 | 60 | 1 | 0 | 0 | 0 | 0 |
| *Andrena hikosana* | solitary | 0 | 0 | 2 | 0 | 0 | 0 | 0 | 0 | 0 | 0 | 0 | 0 | 0 | 0 | 0 | 0 | 0 | 0 |
| *Andrena hondoica* | solitary | 0 | 0 | 0 | 0 | 0 | 0 | 0 | 0 | 2 | 0 | 4 | 3 | 3 | 0 | 0 | 0 | 0 | 0 |
| *Andrena japonica* | solitary | 0 | 2 | 0 | 0 | 0 | 0 | 0 | 0 | 0 | 0 | 0 | 0 | 0 | 0 | 0 | 0 | 0 | 0 |
| *Andrena kaguya* | solitary | 0 | 15 | 7 | 3 | 7 | 6 | 3 | 1 | 2 | 2 | 1 | 3 | 14 | 0 | 1 | 0 | 1 | 0 |
| *Andrena knuthi* | solitary | 0 | 1 | 0 | 0 | 0 | 0 | 0 | 0 | 0 | 0 | 0 | 0 | 0 | 0 | 0 | 0 | 0 | 0 |
| *Andrena komachi* | solitary | 0 | 0 | 0 | 0 | 2 | 0 | 0 | 1 | 0 | 0 | 0 | 0 | 0 | 0 | 0 | 0 | 0 | 0 |
| *Andrena longitibialis* | solitary | 0 | 0 | 0 | 0 | 0 | 0 | 0 | 0 | 0 | 0 | 0 | 0 | 1 | 0 | 0 | 0 | 0 | 0 |
| *Andrena lonicerae* | solitary | 0 | 0 | 1 | 0 | 1 | 1 | 0 | 2 | 0 | 0 | 0 | 0 | 4 | 0 | 0 | 0 | 0 | 0 |
| *Andrena mikado* | solitary | 0 | 0 | 0 | 0 | 0 | 0 | 0 | 0 | 0 | 0 | 0 | 0 | 1 | 0 | 0 | 0 | 0 | 0 |
| *Andrena mitakensis* | solitary | 0 | 0 | 0 | 0 | 0 | 0 | 0 | 0 | 0 | 0 | 2 | 2 | 0 | 0 | 0 | 0 | 0 | 0 |
| *Andrena miyamotoi* | solitary | 2 | 0 | 0 | 0 | 0 | 0 | 0 | 0 | 3 | 0 | 1 | 8 | 2 | 0 | 0 | 0 | 0 | 0 |
| *Andrena munakatai* | solitary | 2 | 94 | 0 | 1 | 4 | 2 | 0 | 5 | 1 | 0 | 5 | 198 | 104 | 0 | 0 | 1 | 0 | 1 |
| *Andrena nawai* | solitary | 0 | 0 | 0 | 0 | 0 | 0 | 0 | 0 | 0 | 0 | 0 | 0 | 5 | 0 | 0 | 0 | 0 | 0 |
| *Andrena omogensis* | solitary | 0 | 0 | 0 | 0 | 0 | 0 | 0 | 0 | 0 | 0 | 1 | 0 | 0 | 0 | 0 | 0 | 0 | 0 |
| *Andrena opacifovea* | solitary | 0 | 0 | 0 | 0 | 0 | 0 | 0 | 0 | 0 | 0 | 0 | 1 | 0 | 0 | 0 | 0 | 0 | 0 |
| *Andrena parathoracica* | solitary | 0 | 0 | 0 | 0 | 0 | 0 | 0 | 0 | 1 | 0 | 0 | 0 | 0 | 0 | 0 | 0 | 0 | 0 |
| *Andrena richardsi* | solitary | 1 | 0 | 1 | 0 | 0 | 0 | 0 | 0 | 0 | 1 | 0 | 1 | 0 | 0 | 0 | 0 | 0 | 0 |
| *Andrena ruficrus* | solitary | 0 | 2 | 0 | 0 | 0 | 0 | 0 | 0 | 1 | 0 | 0 | 0 | 0 | 0 | 0 | 0 | 0 | 0 |
| *Andrena sakagamii* | solitary | 0 | 0 | 0 | 0 | 0 | 2 | 0 | 0 | 0 | 0 | 0 | 0 | 0 | 0 | 0 | 0 | 0 | 0 |
| *Andrena semirugosa* | solitary | 0 | 1 | 0 | 0 | 0 | 0 | 0 | 0 | 0 | 0 | 2 | 5 | 0 | 0 | 0 | 0 | 0 | 0 |
| *Andrena takachihoi* | solitary | 0 | 1 | 0 | 0 | 0 | 0 | 0 | 0 | 0 | 0 | 0 | 0 | 0 | 0 | 0 | 0 | 0 | 0 |
| *Andrena togashii* | solitary | 0 | 5 | 0 | 0 | 1 | 0 | 0 | 0 | 0 | 0 | 0 | 0 | 0 | 0 | 0 | 0 | 0 | 0 |
| *Andrena tsukubana* | solitary | 7 | 0 | 0 | 0 | 0 | 0 | 0 | 0 | 0 | 0 | 0 | 0 | 2 | 0 | 0 | 0 | 0 | 0 |
| *Andrena watasei* | solitary | 0 | 4 | 1 | 2 | 4 | 1 | 0 | 3 | 0 | 1 | 16 | 31 | 31 | 0 | 0 | 0 | 0 | 0 |
| *Andrena yamato* | solitary | 0 | 0 | 0 | 0 | 0 | 0 | 0 | 0 | 0 | 0 | 1 | 1 | 2 | 0 | 0 | 0 | 0 | 0 |
| *Andrena* sp1 | solitary | 2 | 1 | 0 | 0 | 0 | 0 | 0 | 0 | 0 | 0 | 0 | 0 | 0 | 0 | 0 | 0 | 0 | 0 |
| *Andrena* sp2 | solitary | 0 | 0 | 1 | 1 | 0 | 0 | 0 | 0 | 0 | 0 | 0 | 0 | 0 | 0 | 0 | 0 | 0 | 0 |
| *Ceratina esakii* | solitary | 0 | 1 | 1 | 3 | 0 | 2 | 0 | 0 | 0 | 0 | 0 | 8 | 15 | 0 | 0 | 0 | 0 | 0 |
| *Ceratina flavipes* | solitary | 9 | 58 | 0 | 0 | 0 | 0 | 0 | 0 | 0 | 0 | 13 | 0 | 9 | 0 | 0 | 0 | 0 | 0 |
| *Ceratina iwatai* | solitary | 0 | 1 | 0 | 0 | 0 | 0 | 0 | 0 | 0 | 0 | 0 | 0 | 0 | 0 | 0 | 0 | 0 | 0 |
| *Ceratina japonica* | solitary | 19 | 343 | 0 | 2 | 1 | 2 | 0 | 0 | 0 | 0 | 19 | 97 | 99 | 0 | 0 | 1 | 0 | 1 |
| *Ceratina megastigmata* | solitary | 0 | 15 | 0 | 0 | 4 | 22 | 3 | 1 | 0 | 3 | 0 | 9 | 8 | 1 | 1 | 0 | 9 | 0 |
| *Ceratina satoi* | solitary | 0 | 1 | 0 | 0 | 0 | 0 | 0 | 0 | 0 | 0 | 0 | 0 | 0 | 0 | 0 | 0 | 0 | 0 |
| *Colletes babai* | solitary | 0 | 0 | 0 | 0 | 0 | 0 | 0 | 0 | 0 | 0 | 2 | 0 | 0 | 0 | 0 | 0 | 0 | 0 |
| *Colletes esakii* | solitary | 0 | 0 | 0 | 0 | 0 | 0 | 0 | 0 | 0 | 0 | 1 | 0 | 0 | 0 | 0 | 0 | 0 | 0 |
| *Colletes patellatus* | solitary | 1 | 10 | 0 | 0 | 0 | 0 | 0 | 0 | 1 | 0 | 3 | 0 | 0 | 0 | 0 | 0 | 0 | 0 |
| *Colletes perforator* | solitary | 0 | 0 | 0 | 0 | 0 | 0 | 0 | 0 | 0 | 0 | 11 | 0 | 0 | 0 | 0 | 0 | 0 | 0 |
| *Halictus aerarius* | solitary | 0 | 1 | 0 | 0 | 0 | 0 | 0 | 0 | 0 | 0 | 0 | 0 | 0 | 0 | 0 | 0 | 0 | 0 |
| *Hylaeus floralis* | solitary | 4 | 11 | 0 | 0 | 2 | 1 | 3 | 0 | 1 | 2 | 9 | 7 | 1 | 1 | 0 | 1 | 0 | 0 |
| *Hylaeus globula* | solitary | 3 | 5 | 1 | 1 | 0 | 0 | 0 | 0 | 0 | 1 | 0 | 0 | 0 | 0 | 0 | 0 | 0 | 0 |
| *Hylaeus nippon* | solitary | 18 | 11 | 0 | 0 | 3 | 0 | 0 | 0 | 1 | 0 | 10 | 3 | 2 | 0 | 0 | 0 | 0 | 0 |
| *Hylaeus nipponicus* | solitary | 1 | 4 | 0 | 0 | 0 | 0 | 0 | 0 | 0 | 0 | 2 | 0 | 0 | 0 | 0 | 0 | 0 | 0 |
| *Hylaeus pectoralis* | solitary | 1 | 0 | 1 | 0 | 0 | 0 | 0 | 0 | 0 | 0 | 0 | 0 | 0 | 0 | 0 | 0 | 0 | 0 |
| *Hylaeus* sp1 | solitary | 0 | 0 | 0 | 0 | 0 | 0 | 0 | 0 | 0 | 0 | 0 | 1 | 0 | 0 | 0 | 0 | 0 | 0 |
| *Hylaeus* sp2 | solitary | 0 | 0 | 1 | 0 | 2 | 0 | 1 | 0 | 0 | 2 | 0 | 0 | 0 | 0 | 0 | 0 | 0 | 0 |
| *Hylaeus* sp3 | solitary | 0 | 1 | 0 | 0 | 0 | 0 | 0 | 0 | 0 | 0 | 0 | 0 | 0 | 0 | 0 | 0 | 0 | 0 |
| *Lasioglossum affine* | solitary | 0 | 3 | 0 | 0 | 0 | 0 | 0 | 0 | 0 | 0 | 0 | 0 | 0 | 0 | 0 | 0 | 0 | 0 |
| *Lasioglossum allodalum* | solitary | 11 | 0 | 0 | 0 | 0 | 0 | 0 | 0 | 1 | 0 | 0 | 1 | 1 | 0 | 0 | 0 | 0 | 0 |
| *Lasioglossum apristum* | solitary | 18 | 50 | 7 | 2 | 0 | 9 | 0 | 1 | 2 | 0 | 7 | 74 | 3 | 1 | 0 | 0 | 4 | 0 |
| *Lasioglossum baleicum* | solitary | 16 | 2 | 0 | 1 | 8 | 6 | 0 | 1 | 0 | 8 | 2 | 8 | 0 | 0 | 0 | 0 | 0 | 1 |
| *Lasioglossum blakistoni* | solitary | 4 | 0 | 0 | 0 | 0 | 0 | 0 | 0 | 0 | 0 | 19 | 11 | 5 | 0 | 0 | 0 | 0 | 0 |
| *Lasioglossum duplex* | solitary | 0 | 1 | 0 | 0 | 0 | 0 | 0 | 0 | 0 | 0 | 1 | 4 | 1 | 0 | 0 | 0 | 0 | 0 |
| *Lasioglossum ebmerianum* | solitary | 0 | 0 | 0 | 0 | 1 | 1 | 0 | 0 | 0 | 0 | 7 | 3 | 0 | 0 | 0 | 0 | 0 | 0 |
| *Lasioglossum exiliceps* | solitary | 0 | 0 | 0 | 0 | 0 | 0 | 0 | 0 | 0 | 0 | 3 | 13 | 2 | 2 | 1 | 1 | 2 | 0 |
| *Lasioglossum hirashimae* | solitary | 0 | 1 | 0 | 0 | 0 | 0 | 0 | 0 | 0 | 0 | 1 | 0 | 14 | 0 | 0 | 0 | 0 | 0 |
| *Lasioglossum japonicum* | solitary | 3 | 0 | 0 | 0 | 0 | 0 | 1 | 0 | 2 | 0 | 0 | 0 | 0 | 0 | 0 | 0 | 0 | 0 |
| *Lasioglossum kansuense* | solitary | 12 | 26 | 5 | 4 | 7 | 0 | 2 | 4 | 0 | 3 | 43 | 51 | 18 | 0 | 3 | 0 | 26 | 3 |
| *Lasioglossum kuroshio* | solitary | 2 | 4 | 0 | 1 | 1 | 1 | 8 | 3 | 18 | 25 | 4 | 1 | 2 | 0 | 0 | 1 | 0 | 0 |
| *Lasioglossum longifacies* | solitary | 4 | 23 | 3 | 3 | 8 | 5 | 3 | 2 | 25 | 9 | 6 | 16 | 31 | 0 | 0 | 0 | 0 | 4 |
| *Lasioglossum mutilum* | solitary | 0 | 15 | 0 | 1 | 0 | 0 | 0 | 2 | 0 | 0 | 7 | 5 | 1 | 0 | 0 | 0 | 0 | 0 |
| *Lasioglossum occidens* | solitary | 13 | 13 | 0 | 0 | 0 | 0 | 0 | 0 | 0 | 0 | 4 | 1 | 0 | 0 | 0 | 0 | 0 | 0 |
| *Lasioglossum pallilomum* | solitary | 9 | 65 | 0 | 0 | 0 | 0 | 1 | 0 | 0 | 1 | 6 | 7 | 0 | 0 | 0 | 0 | 0 | 0 |
| *Lasioglossum problematicum* | solitary | 1 | 7 | 6 | 5 | 0 | 4 | 1 | 5 | 1 | 1 | 5 | 21 | 35 | 0 | 0 | 0 | 0 | 1 |
| *Lasioglossum proximatum* | solitary | 5 | 38 | 8 | 8 | 11 | 15 | 8 | 13 | 12 | 1 | 33 | 8 | 6 | 1 | 0 | 9 | 1 | 0 |
| *Lasioglossum pumilum* | solitary | 0 | 1 | 0 | 0 | 0 | 0 | 0 | 0 | 0 | 0 | 0 | 0 | 0 | 0 | 0 | 0 | 0 | 0 |
| *Lasioglossum scitulum* | solitary | 5 | 0 | 0 | 0 | 0 | 0 | 0 | 0 | 0 | 0 | 0 | 0 | 0 | 0 | 0 | 0 | 0 | 0 |
| *Lasioglossum sibiriacum* | solitary | 18 | 10 | 0 | 0 | 0 | 0 | 0 | 0 | 0 | 0 | 12 | 9 | 1 | 0 | 0 | 0 | 0 | 0 |
| *Lasioglossum sphecodicolor* | solitary | 0 | 2 | 0 | 0 | 0 | 0 | 0 | 0 | 0 | 0 | 2 | 0 | 3 | 0 | 0 | 0 | 0 | 0 |
| *Lasioglossum transpositum* | solitary | 28 | 48 | 3 | 1 | 5 | 9 | 1 | 0 | 1 | 7 | 20 | 37 | 12 | 2 | 1 | 3 | 4 | 5 |
| *Lasioglossum vulsum* | solitary | 0 | 0 | 0 | 0 | 0 | 0 | 0 | 0 | 0 | 0 | 1 | 0 | 0 | 0 | 0 | 0 | 0 | 0 |
| *Lasioglossum* sp1 | solitary | 0 | 0 | 0 | 0 | 0 | 0 | 0 | 0 | 0 | 0 | 1 | 0 | 0 | 0 | 0 | 0 | 0 | 0 |
| *Lasioglossum* sp2 | solitary | 1 | 0 | 0 | 0 | 0 | 0 | 0 | 0 | 0 | 0 | 0 | 0 | 0 | 0 | 0 | 0 | 0 | 0 |
| *Lasioglossum* sp3 | solitary | 0 | 0 | 0 | 0 | 0 | 0 | 0 | 0 | 0 | 0 | 0 | 1 | 0 | 0 | 0 | 0 | 1 | 0 |
| *Lasioglossum* sp4 | solitary | 0 | 0 | 0 | 0 | 0 | 0 | 8 | 0 | 0 | 1 | 0 | 0 | 0 | 0 | 0 | 0 | 0 | 0 |
| *Macropis tibialis* | solitary | 1 | 0 | 0 | 0 | 0 | 0 | 0 | 0 | 0 | 0 | 0 | 0 | 0 | 0 | 0 | 0 | 0 | 0 |
| *Megachile humilis* | solitary | 0 | 0 | 0 | 1 | 0 | 0 | 0 | 0 | 0 | 0 | 0 | 0 | 0 | 0 | 0 | 0 | 0 | 0 |
| *Megachile remota* | solitary | 0 | 1 | 0 | 0 | 0 | 0 | 0 | 0 | 0 | 0 | 0 | 0 | 0 | 0 | 0 | 0 | 0 | 0 |
| *Megachile sculpturalis* | solitary | 1 | 2 | 0 | 0 | 1 | 0 | 0 | 0 | 0 | 0 | 0 | 0 | 0 | 0 | 0 | 0 | 0 | 0 |
| *Megachile tsurugensis* | solitary | 6 | 15 | 1 | 1 | 0 | 0 | 0 | 0 | 0 | 0 | 6 | 2 | 0 | 0 | 0 | 0 | 0 | 0 |
| *Osmia cornifrons* | solitary | 1 | 0 | 0 | 0 | 0 | 0 | 0 | 0 | 0 | 0 | 0 | 0 | 0 | 0 | 0 | 0 | 0 | 0 |
| *Osmia taurus* | solitary | 3 | 4 | 2 | 0 | 5 | 1 | 0 | 9 | 0 | 0 | 4 | 6 | 1 | 0 | 0 | 0 | 0 | 0 |
| *Tetralonia nipponensis* | solitary | 0 | 3 | 0 | 1 | 1 | 0 | 0 | 2 | 0 | 0 | 9 | 0 | 5 | 0 | 0 | 0 | 0 | 0 |
| *Xylocopa appendiculata* | solitary | 0 | 2 | 0 | 0 | 0 | 0 | 0 | 0 | 0 | 0 | 0 | 0 | 0 | 0 | 0 | 0 | 0 | 0 |
| *Coelioxys hiroba* | cleptoparasitic | 6 | 7 | 0 | 1 | 1 | 0 | 0 | 0 | 0 | 0 | 3 | 0 | 0 | 0 | 0 | 0 | 0 | 0 |
| *Coelioxys yanonis* | cleptoparasitic | 0 | 1 | 0 | 0 | 0 | 0 | 0 | 0 | 0 | 0 | 0 | 1 | 0 | 0 | 0 | 0 | 0 | 0 |
| *Epeolus melectiformis* | cleptoparasitic | 1 | 1 | 1 | 0 | 0 | 0 | 0 | 0 | 0 | 0 | 2 | 1 | 0 | 0 | 0 | 0 | 0 | 0 |
| *Nomada amurensis* | cleptoparasitic | 0 | 0 | 0 | 0 | 0 | 0 | 0 | 0 | 1 | 0 | 7 | 8 | 2 | 0 | 0 | 0 | 0 | 0 |
| *Nomada asozuana* | cleptoparasitic | 0 | 1 | 0 | 0 | 0 | 0 | 0 | 0 | 0 | 0 | 0 | 0 | 0 | 0 | 0 | 0 | 0 | 0 |
| *Nomada aswensis* | cleptoparasitic | 0 | 1 | 0 | 0 | 0 | 0 | 0 | 0 | 0 | 0 | 0 | 0 | 0 | 0 | 0 | 0 | 0 | 0 |
| *Nomada comparata* | cleptoparasitic | 0 | 0 | 0 | 0 | 0 | 0 | 0 | 0 | 0 | 0 | 0 | 1 | 0 | 0 | 0 | 0 | 0 | 0 |
| *Nomada flavoguttata* | cleptoparasitic | 0 | 0 | 0 | 0 | 0 | 0 | 0 | 0 | 0 | 0 | 1 | 6 | 4 | 0 | 0 | 0 | 0 | 0 |
| *Nomada ginran* | cleptoparasitic | 3 | 8 | 2 | 1 | 5 | 1 | 0 | 6 | 0 | 0 | 8 | 4 | 1 | 0 | 0 | 1 | 2 | 0 |
| *Nomada hakonensis* | cleptoparasitic | 1 | 1 | 6 | 0 | 6 | 1 | 0 | 9 | 0 | 0 | 1 | 1 | 2 | 0 | 0 | 0 | 0 | 0 |
| *Nomada harimensis* | cleptoparasitic | 1 | 2 | 0 | 1 | 1 | 0 | 0 | 1 | 0 | 0 | 0 | 23 | 10 | 0 | 0 | 0 | 0 | 0 |
| *Nomada issikii* | cleptoparasitic | 2 | 0 | 0 | 0 | 0 | 0 | 1 | 0 | 0 | 0 | 0 | 0 | 0 | 0 | 0 | 0 | 0 | 0 |
| *Nomada okubira* | cleptoparasitic | 1 | 9 | 0 | 0 | 0 | 0 | 0 | 0 | 1 | 0 | 0 | 97 | 48 | 0 | 0 | 0 | 0 | 0 |
| *Nomada roberjeotiana* | cleptoparasitic | 0 | 0 | 0 | 0 | 0 | 0 | 1 | 0 | 1 | 0 | 0 | 0 | 0 | 0 | 0 | 0 | 0 | 0 |
| *Nomada towada* | cleptoparasitic | 0 | 1 | 0 | 0 | 0 | 0 | 0 | 3 | 0 | 0 | 0 | 1 | 1 | 0 | 0 | 0 | 0 | 0 |
| *Nomada* sp | cleptoparasitic | 0 | 0 | 0 | 0 | 0 | 0 | 2 | 0 | 0 | 0 | 0 | 0 | 0 | 0 | 0 | 0 | 0 | 0 |
| *Sphecodes maruyamanus* | cleptoparasitic | 26 | 19 | 12 | 15 | 2 | 2 | 5 | 0 | 8 | 10 | 3 | 22 | 6 | 0 | 0 | 9 | 1 | 2 |
| *Sphecodes nipponicus* | cleptoparasitic | 11 | 29 | 5 | 6 | 1 | 0 | 1 | 0 | 0 | 0 | 16 | 0 | 2 | 0 | 2 | 0 | 2 | 0 |
| *Sphecodes simillimus* | cleptoparasitic | 3 | 6 | 0 | 3 | 0 | 0 | 0 | 0 | 0 | 0 | 12 | 2 | 2 | 0 | 0 | 0 | 0 | 0 |
| *Sphecodes* sp1 | cleptoparasitic | 1 | 1 | 0 | 0 | 0 | 0 | 0 | 0 | 0 | 0 | 0 | 0 | 0 | 0 | 0 | 0 | 0 | 0 |
| *Sphecodes* sp2 | cleptoparasitic | 0 | 1 | 0 | 0 | 0 | 0 | 0 | 0 | 0 | 0 | 0 | 0 | 0 | 0 | 0 | 0 | 0 | 0 |
